# A comparison of machine learning methods for classification using simulation with multiple real data examples from mental health studies

**DOI:** 10.1177/0962280213502437

**Published:** 2013-09-18

**Authors:** Mizanur Khondoker, Richard Dobson, Caroline Skirrow, Andrew Simmons, Daniel Stahl

**Affiliations:** 1 King's College London, Institute of Psychiatry, Department of Biostatistics, London, UK; 2 King's College London, Institute of Psychiatry, NIHR Biomedical Research Centre for Mental Health at the South London and Maudsley NHS Foundation Trust, London, UK; 3 King's College London, Institute of Psychiatry, NIHR Biomedical Research Unit for Dementia at the South London and Maudsley NHS Foundation Trust, London, UK; 4 King's College London, Institute of Psychiatry, MRC Social, Genetic and Developmental Psychiatry Centre, UK

**Keywords:** machine learning, cross-validation, generalisation error, truncated distribution, microarrays, electroencephalogram (EEG), magnetic resonance imaging (MRI)

## Abstract

**Background:**

Recent literature on the comparison of machine learning methods has raised questions about the neutrality, unbiasedness and utility of many comparative studies. Reporting of results on favourable datasets and sampling error in the estimated performance measures based on single samples are thought to be the major sources of bias in such comparisons. Better performance in one or a few instances does not necessarily imply so on an average or on a population level and simulation studies may be a better alternative for objectively comparing the performances of machine learning algorithms.

**Methods:**

We compare the classification performance of a number of important and widely used machine learning algorithms, namely the Random Forests (RF), Support Vector Machines (SVM), Linear Discriminant Analysis (LDA) and k-Nearest Neighbour (kNN). Using massively parallel processing on high-performance supercomputers, we compare the generalisation errors at various combinations of levels of several factors: number of features, training sample size, biological variation, experimental variation, effect size, replication and correlation between features.

**Results:**

For smaller number of correlated features, number of features not exceeding approximately half the sample size, LDA was found to be the method of choice in terms of average generalisation errors as well as stability (precision) of error estimates. SVM (with RBF kernel) outperforms LDA as well as RF and kNN by a clear margin as the feature set gets larger provided the sample size is not too small (at least 20). The performance of kNN also improves as the number of features grows and outplays that of LDA and RF unless the data variability is too high and/or effect sizes are too small. RF was found to outperform only kNN in some instances where the data are more variable and have smaller effect sizes, in which cases it also provide more stable error estimates than kNN and LDA. Applications to a number of real datasets supported the findings from the simulation study.

## 1 Background

Recent advances in genomic, proteomic, neuroimaging and other high-throughput technologies have led to an explosion of high-dimensional data requiring development of novel methods or modification of existing statistical and machine learning techniques to maximise the information gain from such data. An increase in the number of available methods has logically necessitated method comparisons in order to find the best one in a particular situation resulting in numerous publications focusing on comparative studies in the recent bioinformatics and computational biology literature. A large body of such studies have compared supervised statistical and machine learning methods for subject classification predominantly based on microarray gene expression or high-dimensional mass spectrometry data.^1–10^

Recent literature^11–15^ on the subject has raised questions about the neutrality, unbiasedness, utility and the ways most of these comparisons are performed as there is little consensus between the findings of such studies. A review by Boulesteix et al.^11^ indicated a tendency in some comparative studies to demonstrate the superiority of a particular method using datasets favouring the chosen method. Similar concerns were echoed in a recent *Bioinformatics* paper by Yousefi et al.^15^ suggesting (i) reporting of results on favourable datasets and (ii) the so-called *multiple-rule bias* where multiple classification rules are compared on datasets purporting to show the advantage of a certain method, as the major sources of bias in such comparisons.

There are comparative studies where the objectives are not to demonstrate one particular method as better than the others. Several such studies, the so-called *neutral* comparisons, are cited by Boulesteix et al.^11^ One limitation of these studies is that the comparisons are mainly based on real datasets, and a problem with comparing classification performance estimated on real datasets is the sampling error or noise in the estimated performance measures. Due to the fact that performance estimates are subject to sampling variability,^12,15^ the best performance in one or a few instances does not necessarily imply so on an average or on a population level.

Three alternative routes can potentially be explored for a more robust and objective comparison: (i) using statistical test to take account of the sampling variability or noise in the performance estimates, (ii) comparing analytically based on the distribution theory of the performance estimates and (iii) repeatedly estimating the performance measure on a large number of simulated/synthetic data to average out the sampling variability or noise from the estimated performance criterion. Hanczar and Dougherty^14^ investigated the possibility of using statistical tests for performance comparison and concluded that direct comparison based on statistical test is unreliable and can often lead to wrong conclusions. Analytic comparison would be an elegant approach but requires finding the sampling distribution of the performance estimates based on the joint distribution of feature and class variables. Due to the fact that many of the modern machine learning algorithms are complex (the so-called *black-box* techniques) and often are not based on any underlying statistical model, working out the analytic distributional properties of performance estimates is not possible except for some classical statistically motivated discrimination methods such as the Linear Discriminant Analysis (LDA^16^). As indicated by Hua et al.,^17^ this leaves open the simulation route as a feasible and viable means of objectively studying the characteristics of performance measures based on learning algorithms of a wide range of complexities and forms. This has been realised in other studies in the literature such as Hanczar and Dougherty,^14^ which concluded that ‘the classification rule comparison in real data is worse than in the artificial data experiments. … strongly suggest that researchers make their comparative studies on synthetic datasets’.

We also think that simulation studies are a viable and practical way of figuring out ‘which method performs better in what circumstances’. The *truth* is always known in simulated data and therefore it is easy to investigate bias, the closeness of an estimate to the truth, which is not easy with real data. Synthetic data also make it possible to study the properties of an estimator at varying levels of different data characteristics such as variability, sample size, effect size, correlation, etc. However, the role of real data is also important and should be used to complement the simulation-based investigation as the patterns and structures in real data are generally much more complex and no simulation model can fully capture the patterns, dimensions and sources of variability in data generated from a real biological system.

In this study, we undertake an extensive simulation experiment to compare the classification performance of a number of important and widely used machine learning algorithms ranging from the most classical LDA^16^ to modern methods such as the Support Vector Machines (SVM^18–20^). Although comparisons in the literature have mostly been on real data, synthetic data have also been considered to some extent previously.^3,6,7,14^ However, simulation studies previously used were limited in terms of the number of data characteristics and their coverage (parameter space) considered. The utilities of multi-factorial designs for simulation experiments were discussed by Skrondal^21^ in the context of Monte Carlo experiments. Simultaneous investigation of multiple factors each at multiple levels helps improve the external validity, the extent to which the results can be gerneralised to other situations and to the population, of the conclusions from the simulation study. Using massively parallel processing on high-performance supercomputers (Edinburgh Compute and Data Facility, ECDF, and NIHR Biomedical Research Centre for Mental Health Linux Cluster), we evaluate and compare generalisation errors (leave-one-out cross-validation (CV) errors) for a large number of combinations of the following seven factors: number of variables (*p*), training sample size (*n*), biological (or, between-subjects) variation ($\sigma_{b}$), within-subject variation ($\sigma_{e}$), effect size (fold-change, *θ*), replication (*r*) and correlation (*ρ*) between variables. We believe that there is no *one-size-fits-all* type method in machine learning, which was realised long ago by Wolpert^22^ and reiterated in a recent *Bioinformatics* editorial paper by Rocke et al.^12^ suggesting that there is no classification method that outperforms all others in all circumstances. The motivation of considering such a wide range of factors is to provide some guideline about ‘which method performs better in what circumstances’. We also complement our findings on simulated data by evaluating the performance on a number of real life experimental datasets generated from a range of high-throughput platforms such as gene expression data from DNA microarrays, neuroimaging data from high-resolution magnetic resonance imaging (MRI) system, and event-related potential (ERP) data measuring brain activity derived from electroencephalogram (EEG) system. The simulation program (simData) and performance estimation program (classificationError) are provided as part of the R package optBiomarker available from the Comprehensive R Archive Network (http://www.cran.r-project.org/web/packages/optBiomarker/).

## 2 Methods

### 2.1 Classification methods

We compare classification performance of a number of widely used classification methods based on a diverse range of algorithms and architecture, namely the decision tree and resampling based method, Random Forests, RF^23^; kernel-based learning algorithm, SVM^18–20^; statistically motivated classical method, LDA^24–26^; and instance-based (closest training examples) algorithm k-Nearest Neighbour, kNN.^27,28^

### 2.2 Optimising tuning parameters

Most classification algorithms have their own tuning parameters, which ideally require optimisation on each dataset the methods are applied to. We optimise important user customisable parameters for each method on every simulated dataset using a grid search over supplied parameter spaces. Our search spaces for tuning parameters always included the software default values for the respective parameters to ensure that the performance estimates at optimised parameters are at least as good as that at the default choices.

The RF method is suggested to be quite robust with respect to the variation of its tuning parameters. Leo Breiman (in the manual of original FORTRAN program of RF) suggested that *mtry* (number of variables to be used as candidates at each node) is the only parameter that requires some judgment to set. We optimise *mtry* using a grid search over a random sample of size 5, inclusive of the default value ($\mathrm{mtry}=[\sqrt{p}]$), from the sequence {1,…,[p/2]} where the notation [*x*] represents the floor function, giving the greatest integer not greater than *x*. For p<10, the sequence {1,…,[p/2]} will have less than five elements in which case we run the grid search over the sequence itself. It has been suggested in the literature (e.g., Díaz-Uriarte and Alvarez de Andrés^29^) that there is little need to fine-tune the other parameters of RF for excellent performance. We however considered optimising *nodesize* (minimum size of terminal nodes) and *ntree* (number of trees to grow) in addition to the *mtry* parameter. The parameter *nodesize* controls the length of the trees as no node with fewer cases than the *nodesize* will be split. Smaller node sizes generally give better accuracy and larger node size gives computational advantage for larger datasets typically at the cost of little loss in accuracy. We however run the grid search over the parameter space {1(=default),2,…,5} for each simulated dataset to find the optimal *nodesize*. The parameter *ntree* controls the number of trees to be grown in the forest. Intuitively larger forest (more trees) should be better for stability. Here, we tune *ntree* for optimal accuracy over the parameter space {50,100,500,1000} which includes the randomForest default value (500).

We evaluated SVM with linear, polynomial and radial basis function (RBF) kernels and chose RBF for all calculations as it was found to be less biased than the polynomial kernel in our simulation and also because it is more general than the linear kernel. This is chosen by using the kernel argument (kernel = ”radial”) of the R function svm. The performance of SVM with RBF kernel may depend on the *cost* (the C-constant of the penalty term in the Lagrange multipliers) and *gamma* (the inverse-width parameter of RBF kernel function). The *cost* parameter controls the margin of the support vectors – a smaller value relaxes the penalty on margin errors (ignores penalising points close to the boundary) and hence increases the margin of classification. The value of *gamma* controls the curvature of the decision boundary – higher values make the decision boundary more flexible (non-linear). We optimise the *cost* and *gamma* parameters using grid search over the spaces consisting of uniform samples of size 5 (inclusive of the default values) from the ranges (1/10, 10) and (1/10p,10/p), respectively. It may be noted that the parameter spaces are chosen to span between (1/10)×default and 10×default where the default values for the *cost* and *gamma* parameters are 1 and 1/*p*, respectively. The kNN requires tuning only *k* (the number of nearest neighbours) which we optimise over the range $\left\{1,2,\ldots ,\sqrt{n}\right\}$. We use 10-fold cross-validation, which is computationally less demanding than the leave-one-out cross-validation for optimising all parameters.

### 2.3 Performance estimators

We compare performance of the methods in terms of classification error, sensitivity and specificity. We use leave-one-out cross-validation for estimating these performance measures. This choice was based on evaluation and comparison of several estimators, namely the leave-one-out CV, 10-fold CV, bootstrap and 0.632 plus bootstrap^30^ estimators. The estimators were compared in terms of bias and variability using simulations for training samples of various sizes. We consider training sample sizes (*n*) ranging from 10 to 250, but all four estimators could not be compared for the entire range as bootstrap and 0.632 plus bootstrap estimators often lead to computational problems for smaller *n* due to the extremely unbalanced nature of resampled data. In order to keep bootstrap-based estimators in the comparison, we restricted the sample size to be at least 50 and a comparison based on average performance over 10 different training sample sizes (n=50,60,…,140) shows bootstrap-based estimators to be less biased, but highly unstable even for moderate (n≈50) training sample sizes (data not shown). As we plan to compare methods for a wide range (with *n* as small as 10) of sample sizes, we make a choice between leave-one-out and 10-fold CV estimators. Leave-one-out estimators are supposed to be less biased but more variable for smaller samples than the corresponding 10-fold CV estimators. Our comparison on simulated data shows that leave-one-out estimator has very similar variability to 10-fold CV estimator (see supplementary Figure S1). We finally choose leave-one-out estimator for all comparisons as it is applicable to data of any size and is less biased than the 10-fold CV estimator.

### 2.4 Example datasets

We evaluated the performance of the methods on a number of real datasets generated from a range of high-throughput platforms such as gene expression data from DNA microarrays, neuroimaging data from high-resolution MRI system and ERP data measuring brain activity derived from EEG system.

#### 2.4.1 Bipolar gene expression data

This dataset is based on a microarray gene expression study^31^ of adult postmortem brain tissue (dorsolateral prefrontal cortex) from subjects with bipolar disorder and healthy controls. Affymetrix HG-U133A GeneChips platform was used to determine the expression of approximately 22,000 mRNA transcripts of 61 subjects (30 bipolar and 31 controls). Preprocessed RMA normalised data of this experiment are obtained from GEO (http://www.ncbi.nlm.nih.gov/geo/), accession number GSE5388..

#### 2.4.2 MRI brain imaging data

The brain imaging data were downloaded from the Alzheimer’s disease Neuroimaging Initiative (ADNI) database (www.loni.ucla.edu/ADNI, PI Michael M Weiner). ADNI was launched in 2003 by the National Institute on Aging (NIA), the National Institute of Biomedical Imaging and Bioengineering (NIBIB), the Food and Drug Administration (FDA), private pharmaceutical companies and non-profit organisations, as a $60 million, five-year public–private partnership. The primary goal of ADNI has been to test whether serial MRI, PET and other biological markers are useful in clinical trials of MCI and early Alzheimer’s Disease (AD). Determination of sensitive and specific markers of very early AD progression is intended to aid researchers and clinicians to develop new treatments and monitor their effectiveness, as well as lessen the time and cost of clinical trials. ADNI subjects aged 55 to 90 from over 50 sites across the United States and Canada participated in the research and more detailed information is available at www.adni-info.org. 1.5-T MRI data were downloaded from the ADNI website (www.loni.ucla.edu/ADNI). The description of the data acquisition of the ADNI study can be found at www.loni.ucla.edu/ADNI/research/Cores/index.shtml. Briefly, data from 1.5-T scanners were used with data collected from a variety of MR systems with protocols optimised for each type of scanner. Full brain and skull coverage was required for the MRI datasets and detailed quality control carried out on all MR images according to previously published quality control criteria.^32,33^ We applied the Freesurfer pipeline (version 5.1.0) to the MRI images to produce regional cortical thickness and volumetric measures. All volumetric measures from each subject were normalised by the subject’s intracranial volume, while cortical thickness measures were not normalised and were used in their raw form.^34^

#### 2.4.3 Electroencephalographic data

EEG signals measure voltage fluctuations recorded from electrodes on the scalp, providing an index of brain activity. These data were obtained from 41 adults with a current diagnosis of ADHD (attention deficit hyperactivity disorder) and 47 individuals with no mental health problems.^35^ EEG was recorded during a 3-min resting condition (eyes open) and a cued continuous performance task (CPT-OX; described in detail in McLoughlin et al.^36^). EEG montage and recording, as well as re-referencing, downsampling and ocular artefact rejection procedures were equivalent to those outlined in Tye et al.^37^ Trials with artefacts exceeding 200 μV peak-to-peak in any channel were rejected from the digitally low-pass filtered data (0.1–30 Hz, 50 Hz notch filter, 12 dB/oct). Continuous EEG data were segmented into 2-s intervals and then power spectra were computed using the Fast Fourier Transform with Hanning window. Quantitative measures of EEG spectral power in the major EEG frequency bands were averaged in the respective frequency intervals (*δ*: 0.5–3.5 Hz, *θ*: 4–7.5 Hz, *α*: 7.5–12.5 and *β*: 12.5–30 Hz). Absolute power density ($\mu V^{2}/\mathrm{Hz}$), relative power density (the proportion of each individual frequency domain contributing to the summed power density) and θ/β ratios were calculated across averaged frontal, central and parietal scalp electrode locations. ERPs were extracted from the CPT-OX as described in McLoughlin et al.^36^

### 2.5 Simulation

#### 2.5.1 Main simulation

Classification problems based on high-dimensional data are predominantly demonstrated using microarray gene expression data. We therefore design our simulation model to generate realistic gene expression data where we can systematically vary different data characteristics (variability, effect size, correlation, etc.) in order to investigate the effects of such data characteristics on the performance of classification algorithms. To make our simulated data as realistic as possible, we base our simulation study on a real microarray dataset. We define a set of base expressions (*μ*) by averaging normalised ${\text{log}}_{2}$ expression data over 28 human blood samples taken from 28 healthy individuals.^38^ The base expression set contains gene expressions for 54,359 markers assayed using CodeLink human whole genome microarray gene expression platform. Systematic variability and stochastic noise in the base expression set can be assumed minimal as the data are normalised and averaged over many individuals. It is therefore reasonable to assume that the base expression levels (*μ*) are proportional to the true mRNA abundance signal of the corresponding markers. We then use a random effects model to introduce pre-specified amount of stochastic noise in the data. We consider two levels of stochastic variability: between-subjects and ^within-subject variations which are more commonly known as *biological* and *technical* variations in the microarray literature. For a given training sample size (*n*), we initially simulate data for each marker independently according to

(1) $$x_{\mathrm{ij}}=\mu +b_{i}+\varepsilon_{\mathrm{ij}},$$

where *x_ij_* denotes the simulated ${\text{log}}_{2}$ expression value for a marker in the *j*th replicate (j=1,2,…,r) of the *i*th subject (i=1,2,…,n). We initially simulate each replicate of every feature as independent (uncorrelated) variable which can be treated as univariate. Data on each marker are then averaged over replicates and a multivariate structure is introduced to the data from multiple (*p*) markers by imposing a *p*-dimensional covariance structure via Cholesky root transformation (see below). The parameter *μ* denotes the base expression (randomly taken from the base expression set), *b_i_* is the random effect for the *i*th biological subject and $\varepsilon_{\mathrm{ij}}$ is the random experimental noise. We assume that *b_i_* and $\varepsilon_{\mathrm{ij}}$ are independent random variables distributed according to $N(0,\sigma_{b}^{2})$ and $N(0,\sigma_{e}^{2})$, respectively. Model (1) allows generating independent or uncorrelated gene expression data with different amount of stochastic noise controlled by the parameters $\sigma_{b}$ and $\sigma_{e}$.

Independence is rarely a realistic assumption for any multidimensional data including gene expressions where groups of genes operate together in an orchestrated fashion forming network relationships to perform certain biological functions. Variables within such groups or networks are generally highly correlated within themselves but are likely to exhibit negligible correlations with variables from another group. We consider a block-diagonal correlation matrix to model such network relationships using the *hub-Toeplitz*^39^ correlation structure for the *h*th block:

(2) $$R_{h}=(1\rho_{h,2}\rho_{h,3}\rho_{h,4}\ldots \rho_{h,d_{h}}\rho_{h,2}1\rho_{h,2}\rho_{h,3}\ldots \rho_{h,d_{h}-1}\rho_{h,3}\rho_{h,2}1\rho_{h,2}\ldots \rho_{h,d_{h}-2}\rho_{h,4}\rho_{h,3}\rho_{h,2}1\ldots \rho_{h,d_{h}-3}\rho_{h,d_{h}}\rho_{h,d_{h}-1}\rho_{h,d_{h}-2}\rho_{h,d_{h}-3}\ldots 1);\;h=1,2,\ldots ,H$$

where

(3) $$\rho h,l=\rho \max+(\frac{l-2}{dh-2})\nu (\rho \max-\rho \min),$$

which decreases from $\rho_{\max}$ to $\rho_{\min}$ for $2\leq l\leq d_{h}$. The parameter *ν* controls the decline rate which is linear for ν=1. The hub-Toeplitz correlation structure assumes a known correlation between a network hub (typically the first variable) and each of the other variables within the block where the correlation between the hub and the *l*th variable decays as *l* increases. This is a more realistic assumption than a block-diagonal correlation matrix with exchangeable structure within blocks considered by Hua et al.^17^

We set the number of blocks (*H*) to 1 for p<5. For p≥5, the number of blocks is randomly selected from the set {1,2,…,[p/3]} where [*x*] represents the floor function (the largest integer not greater than *x*). If *p* is a multiple of *H*, all blocks are considered to have the same dimension, $d_{h}=p/H$. If *p* is not a multiple of *H*, one of the blocks (typically the first) is considered to have dimension $d_{1}=[p/H]+\mathrm{mod}(p,H)$ and $d_{h}=[p/H]$ for the rest (h=2,3,…,H) where mod(p,H) represents the reminder of the division *p*/*H*.

We use Cholesky root transformation to impose the block-diagonal hub-Toeplitz structure $R=\mathrm{diag}\{R_{1},R_{2},\ldots ,R_{H}\}$ to the uncorrelated data. Suppose $\bar{X}=(\bar{X_{1}},\ldots ,\bar{X_{p}})$ is a *p*-vector of random variables representing the averages of the markers over their respective replicates. Then the covariance matrix of $\bar{X}$ is given by

(4) $$V=(\sigma_{b}^{2}+\sigma_{e}^{2}/r)I_{p},$$

where *I_p_* is a *p*-dimensional identity matrix. We then compute the Cholesky root *C* of $V^{\frac{1}{2}}{\mathrm{RV}}^{\frac{1}{2}}$, and the transformed data with the desired covariance structure is obtained as $\bar{Y}=\bar{X}C$.

In order to introduce a systematic source of variation (namely the group difference), we divide the training sample into two groups, *G*1 and *G*2, of sizes $n_{1}=[n/2]$ and $n_{2}=n-n_{1}$, respectively. The groups are then made different by an effect size *δ*, where *δ* represents the unstandardised group difference (${\text{log}}_{2}(\text{foldchange})$). That is, we add a quantity zδ to the simulated expression values in *G*2 to make the data in *G*2 to be up- or down-regulated depending on the value of *z* randomly selected from {-1,1}. We generate *δ* from a truncated normal distribution with support A=[α,∞),α≥0. That is, *δ* follows a truncated normal distribution given by,

(5) $$f(\delta |A)=\frac{\frac{1}{\tau }\varphi (\frac{\delta }{\tau })}{1-\Phi (\frac{\alpha }{\tau })},\;\alpha \leq \delta <\infty .$$

where φ(.) and Φ(.) denote the density and distribution function, respectively, of a standard normal variate and *τ* is the scale of the original (untruncated) zero mean normal variate. The threshold *α* ensures that markers we include in the analysis have non-zero effect sizes. We term $\theta_{\min}=2^{\alpha }$ as the minimum fold change of the markers to be considered in classification. The expected value of *δ* in equation (5) is given by

(6) $$E[\delta |A]=\tau \frac{\varphi (\alpha )}{1-\Phi (\alpha )}.$$

The average fold change (*θ*) of the biomarkers included in the classification can be calculated from $\theta =2^{\tau \frac{\varphi (\alpha )}{1-\Phi (\alpha )}}$ as a function of (α,τ). We intend to investigate the effects of training sample size (*n*), feature set size (*p*), biological variation ($\sigma_{b}$), experimental variation ($\sigma_{e}$), effect size (*θ*), replication (*r*) and correlation (*ρ*) between variables. We consider feature sets of sizes 5, 25, 50, 75 and 100. This should be a reasonable range to understand the patterns of the effect of feature set size on the classification performance. Although high-throughput data can have many more variables, typical goal in classification with such data would be to achieve a good class prediction performance with the smallest possible number of features, termed the optimal number.^40^ The preventing factor for considering larger feature set is the computing cost which increases considerably for every additional variable in the feature set. The values of other factors considered in our simulation are summarised in Table 1. We randomly select $\rho_{\max}$ and $\rho_{\min}$ from the uniform distributions U(0.6,0.8) and U(0.2,0.4), respectively, and set ν=1 for each simulated dataset. We use τ=1 in all simulations and the biomarkers are added in descending order of effect size. All simulations were repeated 500 times, and average error rates were calculated over these 500 datasets for each combination of the levels of the factors.

**Table 1.** The values of training sample size (n), biological variation ($\sigma_{b}$), experimental variation ($\sigma_{e}$), minimum fold change ($\theta_{\min}$), and replication (r) considered in simulation.

Data characteristics (factors)				
*n*	$\sigma_{b}$	$\sigma_{e}$	$\theta_{\min}(\theta )$	*r*
10	0.1	0.1	1.0 (1.74)	1
20	0.5	0.5	1.5 (2.30)	3
30	1.0	1.0	2.0 (2.88)	5
40	1.5	1.5	2.5 (3.45)	7
50	2.0	2.0	3.0 (4.03)	9
75	2.5	2.5	3.5 (4.61)	11
100	3.0	3.0	4.0 (5.18)	13
150	3.5	3.5	4.5 (5.75)	15
200	4.0	4.4	5.0 (6.33)	17

#### 2.5.2 Non-normal data

We perform a sensitivity analysis to investigate possible consequences of departure from normally distributed data. We simulated data from the Poisson family for this sensitivity analysis. Poisson data differ from its Gaussian counterpart in several respects: (i) Poisson is a discrete distribution which generates integer valued data rather than interval scaled (Gaussian like) data, (ii) mean–variance relationship for Poisson data is more restricted than that for Gaussian data as they are identical to the intensity rate parameter (*λ*) and (iii) Poisson data are generally positively skewed, particularly for smaller *λ*. It is not possible to exactly match the mean and variance of Poisson data with their Gaussian counterparts, but we maintained similar (*hub-Toeplitz*) block diagonal covariance structure between variables as that for the Gaussian data and conducted the simulation at various combinations of training sample and feature set sizes (n=10,20,30,40,50,75,100,150,200; p=5,25,50,75,100). We set λ=4 for simulating a single replicate of each variable and introduce systematic group difference (*δ*) in the same way as that for Gaussian data. The choice of *λ* was made as a compromise between asymmetry and data variability: λ=4 is large enough to ensure reasonable data variability and at the same time small enough to ensure skewness of the distribution.

## 3 Results and discussion

### 3.1 Results on main simulation

**Figure 1.**
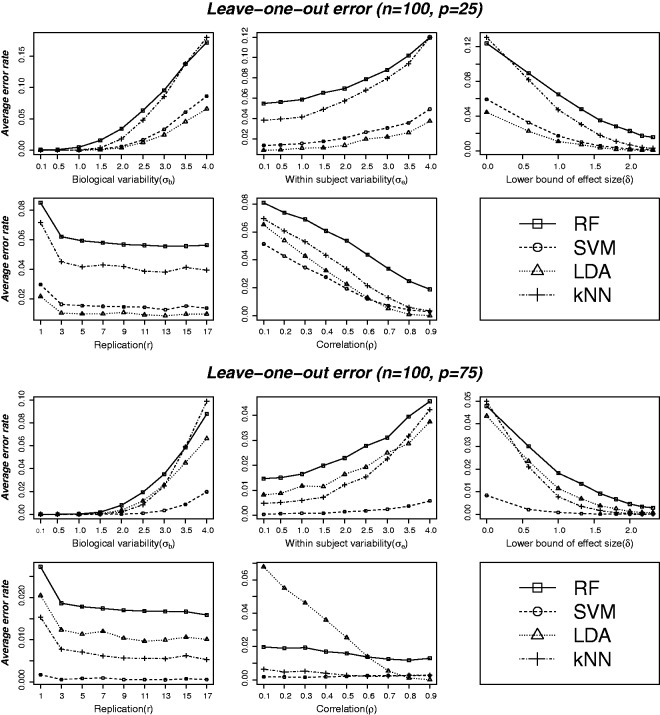
Average leave-one-out cross-validation error at varying levels of different data characteristics: biological variability ($\sigma_{b})$, experimental noise ($\sigma_{e}$), lower bound of effect size (*δ*), replication (r) and correlation between variables (*ρ*). Top and bottom panels correspond to feature set sizes of 25 and 75 variables, respectively, and a common training sample size (*n* = 100). Each plot compares error rates for the four methods at varying levels of a particular parameter as shown on the *x*-axis for given values of the other parameters. The given values are selected from the set ($n=100,\;\sigma_{b}=2.5,\;\sigma_{e}=1.5,\;\theta_{\min}=2,\;r=3$), the correlation structure being of the hub-Toeplitz form (except for the plots against *ρ*, which are based on single-block exchangeable correlation matrix to make the plot against *ρ* meaningful). For smaller feature set (*p* = 25) and higher correlation (ρ>0.6), LDA seems to have performed uniformly better across all levels of the data characteristics considered. SVM outperformed all other methods including LDA for larger feature sets (*p* = 75) and kNN was found to be the second best at this level of feature set size. RF was found to underperform in most scenario except for very noisy data (having high variability and smaller effect size) in which cases it performed better than kNN.

**Figure 2.**
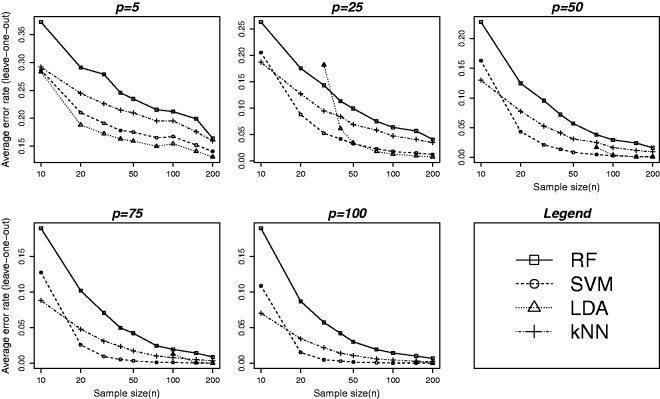
Average leave-one-out cross-validation error at varying levels of training sample and feature set sizes. Error rates are plotted against nine different values of *n* as given in Table 1. The five plots correspond to five different values of *p* (feature set size): 5, 25, 50, 75 and 100, respectively. All other parameters are set to fixed values: ($\sigma_{b}=2.5,\;\sigma_{e}=1.5,\;\theta_{\min}=2,\;r=3,\;\rho =0$). Although the performance of SVM was found to be better than the other methods (see Figure 1) for larger training samples and feature sets, the method does not perform well for smaller (n<20) samples. The plot suggests that the sample size should be at least 20 for SVM to have its superior performance. Error rates for LDA are not shown for sample sizes smaller than the number of variables (*p*) as the method is degenerate for p>n. Although LDA is theoretically valid for any n>p, it can perform very poorly when the feature set size is very close to the training sample size. The plot suggests that *n* should be at least as big as 2p for LDA to have comparable performance.

Average leave-one-out cross-validation estimate of classification error over the 500 replications of simulated datasets for all the four methods (RF, SVM, LDA and kNN) are plotted against the values of the various data characteristics in Figures 1 and 2. Top and bottom panels in Figure 1 are based on (n=100,p=25) and (n=100,p=75), respectively. Error rates for various combinations of these two factors (training sample size and feature set size) are displayed in Figure 2. Results in Figure 1 and the additional plots in the supplementary file (Figure S2) suggest that optimal performance conditions for LDA, in which case it outperforms all the other methods studied here, are smaller feature set size (relative to training sample size) and higher correlation. The region of strength of this method appears to be p/n<0.5 (number of features smaller than approximately half the sample size) and higher than moderate correlation (ρ>0.6) between features. As the feature set gets larger (p/n>0.5), SVM outplays LDA and also performs better than RF and kNN. The bottom panel of Figure 1 compares the average leave-one-out error for *n* = 100 and *p* = 75, i.e., p/n=0.75 showing SVM outperforming the other methods by a clear margin. The margin of performance differences is higher at higher data variability, smaller effect size and smaller correlation. The performance of kNN also improves as the feature set size grows and outperforms LDA and RF unless the data variability is too high and/or effect size is too small. RF was found to outperform only kNN in some instances where the data are more variable and have smaller effect sizes, in which cases it also provides more stable error estimates than kNN and LDA (see supplementary Figure S3).

Corresponding plots for sensitivity and specificity are presented in supplementary Figures S4 and S5. The performances of all the studied methods were found to be symmetric in terms of sensitivity and specificity, which is expected to be the case for balanced and symmetrically distributed data. The patterns and order of performance in terms of sensitivity and specificity were found to be similar to that of overall classification error.

Figure 2 shows error rates plotted against nine different values of *n* as given in Table 1. The five plots correspond to five different values of *p* (feature set size): 5, 25, 50, 75 and 100, respectively. Although SVM was found to perform better than the other methods (see Figure 1) for larger training samples and feature sets, the method does not perform well for smaller (n<20) samples. The plot suggests that the sample size should be at least 20 irrespective of the number of variables (*p*) for SVM to have better performance. The error rates for LDA are not shown for sample sizes smaller than the number of variables (*p*) as the method is degenerate for p>n. Although LDA is theoretically valid for any n>p, it can perform poorly when the feature set size is very close to the training sample size. The plot suggests that *n* should be at least as big as 2p for LDA to have comparable performance. This finding is consistent with that we found earlier.^40^

**Figure 3.**
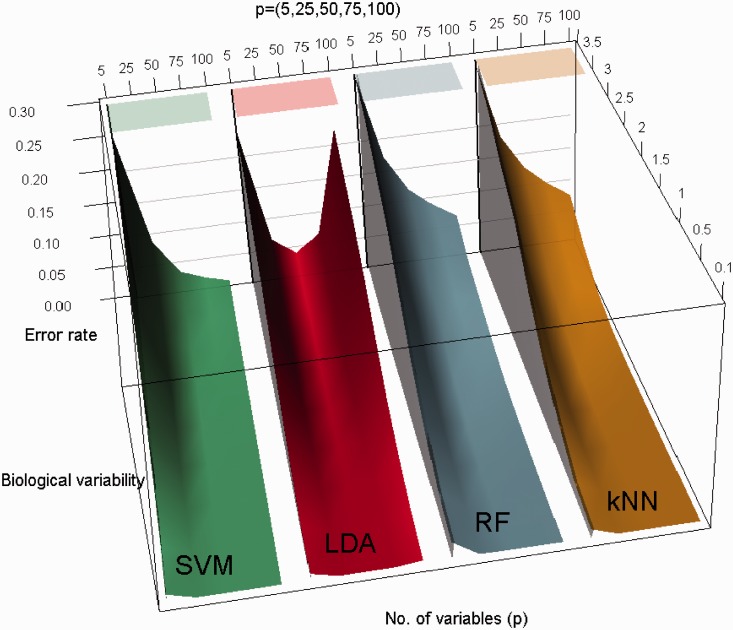
Three-dimensional plot of average leave-one-out cross-validation error as a joint function of feature set size (number of variables) and biological variation. All the plots corresponds to *n* = 100 and the five different feature set size (p=5,25,50,75,100) as shown on header of the plot. The remaining parameters are set to fixed values as indicated in the two-dimensional plots. The plots suggest that the error rate declines as the feature set size (*p*) grows for all methods except LDA. As seen previously in the two-dimensional plots, error rate for LDA starts to increase as the number of variables *p* exceeds beyond n/2. Although SVM outperforms LDA for larger feature sets, LDA performs better than SVM for smaller feature set size (p<20).

To better understand the patterns of classification performance and for the ease of visual comparisons, three-dimensional plots of average leave-one-out cross-validation error as a joint function of feature set size and biological variation are displayed in Figure 3. All the plots correspond to *n* = 100 and the feature set size (*p*) ranges between 5 and 100 inclusive. The figure shows the error rate as a joint function of ($\sigma_{b},p$) and suggests that the error rate declines as the feature set size (*p*) grows for all methods except LDA. As seen previously in the two-dimensional plots, performance of LDA starts to deteriorate as the number of variables *p* exceeds approximately half the sample size (n/2). LDA, however, outperforms SVM for smaller (<0.5) feature set to sample size ratio and vice versa.

The standard errors (SE) of leave-one-out cross-validation error estimates at varying levels of different data characteristics are plotted in supplementary Figure S3. The SEs of error estimates appear to follow very similar patterns to that of average error estimates. For example, SVM provides the most stable (lowest SE) estimates of leave-one-out error in situations where the feature sets are larger than half the size of training samples (p/n>0.5), whereas LDA was found to give most precise estimates of error rates for p/n<0.5 and higher correlations between features. LDA however appears to be least stable for larger feature set size to sample size ratio (p/n>0.5). The strength of RF is visible in situations where data are more variable and have smaller effect sizes, in which cases it provides more stable error estimates than kNN and LDA.

#### 3.1.1 Results on non-normal data

This section presents the results for simulation based on non-normal (Poisson) distributions as described in the simulation section. Supplementary Figure S6 shows average leave-one-out cross-validation estimate of classification error over the 500 replications of simulated data for all the four methods (RF, SVM, LDA and kNN) at various combinations of feature set and training sample size. The patterns and order of performance for Poisson data look very similar to that we observed for Gaussian data (Figure 2). For example, like Gaussian data, SVM is seen not to perform well for smaller samples (n<20) although it outperforms the other methods for larger feature sets with bigger samples. And although LDA can theoretically handle feature set size as large as the sample size (p≈n), its performance seem to deteriorate as the feature set size (*p*) exceeds beyond approximately half the sample size (e.g., see the plot for *p* = 25). Of the four methods compared, only LDA is based on normality assumption, but the others do not rely on any distributional assumption. These results indicate that LDA is robust against some departure from normality and the other methods perform similarly on normal and non-normal data.

### 3.2 Results on real data

Real life data are generally much more complex than the simulated data in terms of patterns and sources of variability. In order to be able to generalise the conclusions, it is important to investigate whether the findings on simulated data are supported by that from real data. We therefore compare the performance of the methods on several real datasets generated from various high-throughput technologies such as gene expression data from DNA microarrays, neuroimaging data from high-resolution MRI system and ERP data measuring brain activity derived from EEG system. Our example datasets are mainly from studies in mental health research, but the findings should be generalisable to any other disease or condition.

#### 3.2.1 Bipolar gene expression data

**Figure 4.**
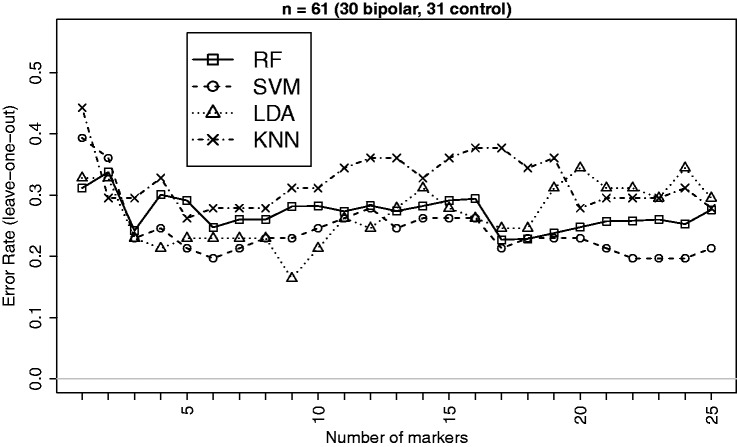
Leave-one-out CV errors for classifying 61 patients into Bipolar and Control groups based on top differentially expressed genes from the bipolar gene expression data.^31^

This dataset is based on a microarray gene expression study^31^ of adult postmortem brain tissue (dorsolateral prefrontal cortex) from subjects with bipolar disorder and healthy controls. More details can be found in the methods section. We select top 25 markers based on the *p*-values of the Empirical Bayes test^41^ for differential expression between the bipolar and control groups for classification analysis. This selection is made in order to avoid using variables that are just noise and have no class discriminatory signals. The same approach is used for all other example datasets we used in this paper. The summary characteristics of bipolar gene expression data based on the selected markers are given in Table 2. Estimated leave-one-out cross validation errors for bipolar versus control classification based on the top 25 markers are displayed in Figure 4.

**Table 2.** Summary of estimated data characteristics for a subset of top 25 markers from the bipolar gene expression data [31].

Group	Measure	Min	Mean	Med	Max
Bipolar	Variation* ($\overset{\wedge }{\sigma }$)	0.14	0.36	0.32	0.91
Correlation ($\overset{\wedge }{\rho }$)	−0.68	0.27	0.40	0.90
Control	Variation ($\overset{\wedge }{\sigma }$)	0.14	0.27	0.21	0.49
Correlation ($\overset{\wedge }{\rho }$)	−0.62	0.18	0.24	0.84
Combined	Variation ($\overset{\wedge }{\sigma }$)	0.14	0.32	0.30	0.72
Correlation ($\overset{\wedge }{\rho }$)	−0.63	−0.18	0.23	0.84
Bipolar vs. Control	Log-ratio ($\left|\overset{\wedge }{\delta }\right|$)	0.16	0.34	0.30	0.85

* Biological variation ($\sigma_{b}$) and technical/experimental variation ($\sigma_{e}$) cannot be estimated separately for these data as there is no replication (*r* = 1). Table shows the combined variability: $\overset{\wedge }{\sigma }=\sqrt{{\overset{\wedge }{\sigma }}_{b}^{2}+{\overset{\wedge }{\sigma }}_{e}^{2}}$.

Effect sizes are not that big for this dataset and the classification performance does not appear to improve as the number of markers grows beyond around 5. However, in terms of comparative performance SVM appears better (closely followed by LDA) in most instances which is consistent with the findings on simulated data. It may be noted that the performance of LDA started to deteriorate when the number of markers exceeded 18 which is much smaller than the threshold (n/2≈30) predicted from simulation studies. This may be due to smaller effect sizes of the markers that can be easily dominated by noise, which is also supported by the fact that RF performed better than kNN as we found with the simulated data of this type.

#### 3.2.2 Brain imaging data of Alzheimer’s and control patients

**Figure 5.**
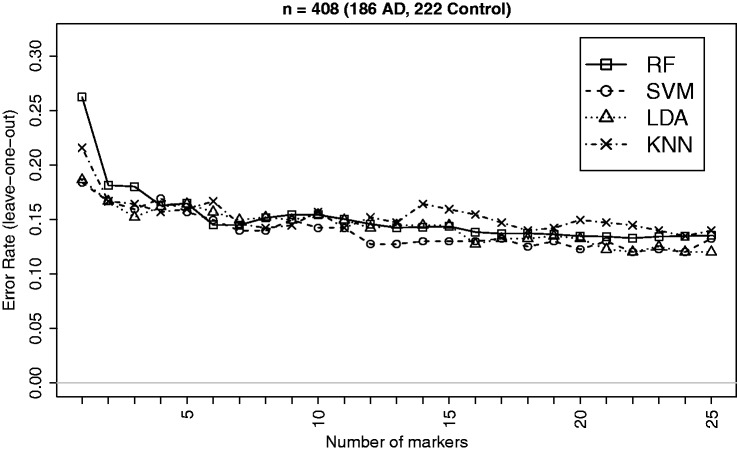
Leave-one-out CV errors for classifying 408 patients into AD and Control groups based on top imaging measures. There is very little differences between the performances of the methods, which is expected due to larger effect sizes and is supported by the findings from simulation indicating that the performance differences gets narrower as the effect size increases.

Recent advances in neuroimaging technologies such as the high-resolution MRI system have made it possible to effectively measure brain-wide regional cortical thickness and regional volume using automated atlas-based neuroimage segmentation scheme. Such measures are commonly used for linking Alzheimer’s disease to the physical changes in different brain regions, e.g., hippocampus and entorhinal cortex. In this study, we consider brain imaging data from the US-based ADNI study (www.loni.ucla.edu/ADNI) for classifying patients on the basis of their regional cortical thickness and volume measures. Further information on the data and the protocols for generating such measures can be found in the method section. We select the top 25 measures on a sample of 418 (186 AD, 222 Controls) patients based on the *p*-values of the Empirical Bayes test^41^ for difference in means between the AD and control (CTR) groups for classification analysis. The summary characteristics of imaging data based on the selected markers are given in Table 3. The classification performances of the four methods (RF, SVM, LDA and kNN) for the AD versus CTR classification are displayed in Figure 5. Effect sizes (*δ*) are higher for this dataset and the performances of the methods appear very close to each other. This is expected and supported by the findings from simulation (e.g., Figure 1) indicating that the performance differences gets narrower as the effect size increases. Overall, SVM and LDA seem to have performed better in most instances as supported by the general patterns observed in simulation.

**Table 3.** Summary of estimated data characteristics for a subset of top 25 brain imaging markers from the ADNI study.

Group	Measure	Summary of estimates			
		Min	Mean	Med	Max
AD	Variation* ($\overset{\wedge }{\sigma }$)	0.78	1.01	1.01	1.17
	Correlation ($\overset{\wedge }{\rho }$)	−0.54	0.16	0.21	0.78
Control	Variation ($\overset{\wedge }{\sigma }$)	0.56	0.86	0.89	1.01
	Correlation ($\overset{\wedge }{\rho }$)	−0.52	0.14	0.13	0.72
Combined	Variation ($\overset{\wedge }{\sigma }$)	0.78	0.93	0.95	1.00
	Correlation ($\overset{\wedge }{\rho }$)	−0.51	0.15	0.18	0.72
AD vs. Control	$\left|\overset{\wedge }{\delta }\right|$	0.76	0.97	0.92	1.26

* See footnote of Table 2.

#### 3.2.3 Electroencephalographic data

EEG signals measure voltage fluctuations recorded from electrodes on the scalp, providing an index of brain activity. EEG data for this example were obtained from 41 adults with a current diagnosis of ADHD (attention deficit hyperactivity disorder) and 47 individuals with no mental health problems.^35^ More detail data description is in the method section. Out of 63 measures, only few were significantly different between case and control groups. We, however, select the top 25 measures based on the *p*-values of the Empirical Bayes test^41^ for classification analysis.

**Figure 6.**
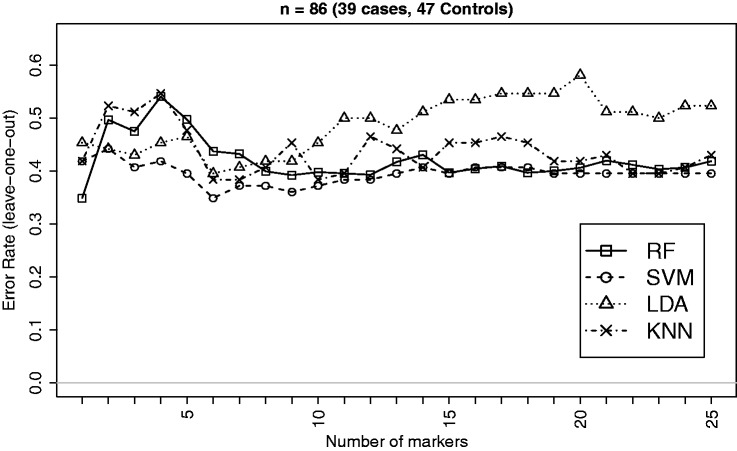
Leave-one-out CV errors for classifying 86 patients into ADHD and Control groups based on top EEG measures. This is an example where the effect size is very small and therefore the error rate does not show a steady declining pattern as the number of variables grows. This however supports the observation from simulation regarding the strengths of RF in dealing with data having weaker signal and higher noise as RF is seen to outperform LDA and kNN in this example.

The summary characteristics of EEG data based on the selected measures on 86 subjects (two ADHD cases excluded due to missing values in some of the selected variables) are given in Table 4. The classification performances of the four methods (RF, SVM, LDA and kNN) for the *ADHD* versus *Control* classification are displayed in Figure 6.

**Table 4.** Summary of estimated data characteristics for a subset of top 25 features from the EEG data.

Group	Measure	Summary of estimates			
		Min	Mean	Med	Max
ADHD	Variation* ($\overset{\wedge }{\sigma }$)	0.74	0.93	0.93	1.12
	Correlation ($\overset{\wedge }{\rho }$)	−0.90	0.07	0.04	0.96
Control	Variation ($\overset{\wedge }{\sigma }$)	0.88	1.05	1.06	1.17
	Correlation ($\overset{\wedge }{\rho }$)	−0.91	0.11	0.04	0.97
Combined	Variation ($\overset{\wedge }{\sigma }$)	0.98	0.99	0.99	1.00
	Correlation ($\overset{\wedge }{\rho }$)	−0.87	0.09	0.03	0.95
ADHD vs. Control	$\left|\overset{\wedge }{\delta }\right|$	0.18	0.28	0.27	0.45

* See footnote of Table 2.

Of the three example datasets, EEG data have the smallest signal (mean $\left|\overset{\wedge }{\delta }\right|$ = 0.28) to discriminate between the case and control groups. There are hardly any EEG measures that differ significantly between the groups. This probably explains why the error curves do not show a steady decline as the number of features grows. RF, however, is seen to outperform LDA and kNN supporting the observation from simulation regarding the strengths of RF in dealing with data having weaker signal and higher noise.

## 4 Conclusions

We performed an extensive simulation study in order to objectively compare classification performance of a number of widely used machine learning or statistical algorithms in terms of generalisation errors, sensitivity and specificity for supervised classification problems. The main focus of our study was to investigate ‘which method performs better in what circumstances’ by comparing performances at various combinations of levels of multiple factors (data characteristics). Results of our simulation study and subsequent examples on multiple real datasets from various high-throughput technology platforms led to the following conclusions:

- For smaller number of correlated features, number of features not exceeding approximately half the sample size, LDA was found to be the method of choice in terms of average generalisation errors as well as stability (precision) of error estimates. The region of strength of LDA appears to be p/n<0.5 (number of features smaller than approximately half the sample size) and higher correlation (ρ>0.6) between features.
- As the feature set gets larger (p/n≥0.5) SVM (with RBF kernel) outplays LDA and also performs better than RF and kNN by a clear margin. The margin of performance differences is higher at higher data variability, smaller effect size and smaller correlation. However, the sample size should be at least 20 irrespective of the number of features (*p*) for SVM to achieve its superior performance.
- The performance of kNN also improves as the feature set size grows and outplays that of LDA and RF unless the data variability is too high and/or effect sizes are too small.
- RF was found to outperform only kNN in some instances where the data were more variable and had smaller effect sizes, in which cases it also provided more stable error estimates than kNN and LDA.
- All methods showed a tendency to perform better at higher correlation, but RF appears to have comparatively worse performance when the variables are very highly correlated.
- Performances of all the studied methods were found to be symmetric in terms of sensitivity and specificity, which is expected to be the case for balanced and symmetrically distributed data.

None of the methods studied (except LDA) require the data to follow any particular probability distribution and the simulation results should be robust against departures from normality assumption. We demonstrated this by simulating from non-normal (Poisson) distribution which indicates that LDA is robust against some departure from normality and the other methods perform similarly on normal and non-normal data.

## Supplementary Material

Supplementary material
