# Supplementary material for: A comparison of machine learning methods for classification using simulation with multiple real data examples from mental health studies
Source: Stat Methods Med Res. 2013 Sep 18;25(5):1804–23. doi: 10.1177/0962280213502437 (PMC5081132; doi:10.1177/0962280213502437)
Supplement: Supplementary material [file supplementary_khondoker.pdf]

Supplementary file for:

A comparison of machine learning methods for classification using simulation with multiple real data examples from mental health studies

Mizanur Khondoker, Richard Dobson, Caroline Skirrow, Andrew Simmons, Daniel Stahl

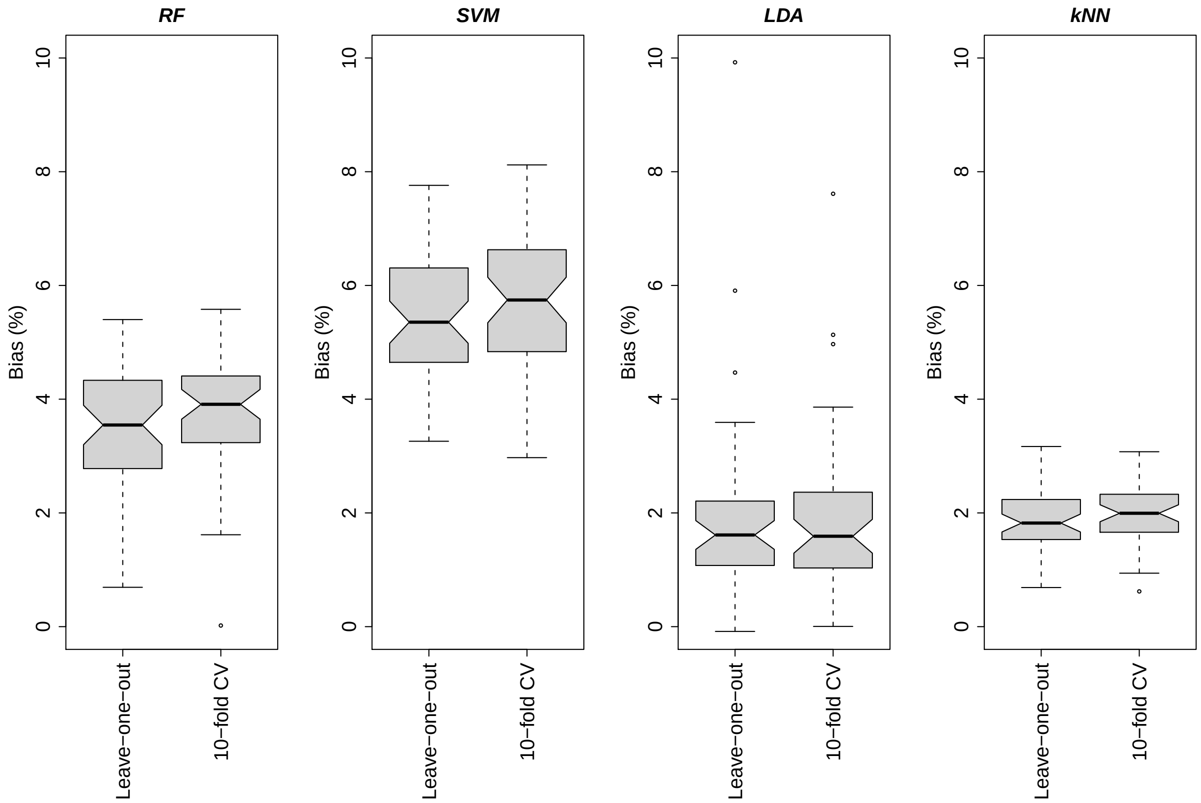

Figure S1: Boxplots of bias as % of true error (0.50) in leave-one-out and 10-fold cv error rates for classifying two groups of observations with no systematic differences between the groups. All estimates are average over 500 repeats of simulated datasets. Data were simulated form normal distribution according to model (1) with a training set size 100 (50 in each group). Each of the box plots shows the distribution of bias for various number of variables (1 to 50) considered in classifying the subjects.

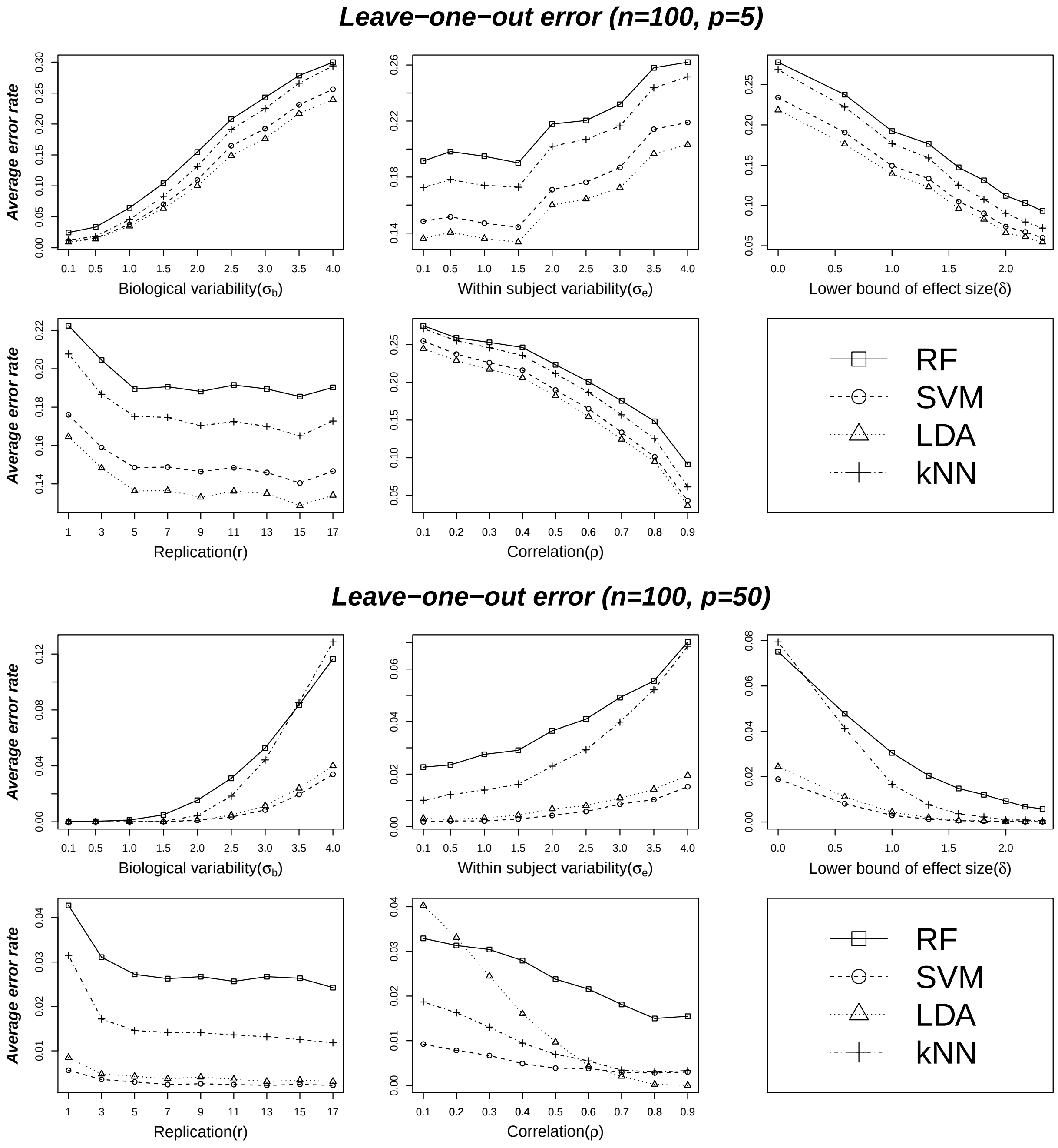

Figure S2: Average leave-one-out cross-validation error at varying levels of different data characteristics: biological variability(σ_b), experimental noise (σ_e), lower bound of effect size (δ), replication (r), and correlation between variables (ρ). Top and bottom panels correspond to feature set sizes of 5 and 50 variables respectively and a common training set size (n=100). Each plot compares error rates for the four methods at varying levels of a particular parameter as shown on the x-axis for given values of the other parameters. The given values are selected from the set ($n = 100, \sigma_b = 2.5, \sigma_e = 1.5, \theta_{min} = 2, r = 3$), the correlation structure being of the hub-Toeplitz form (except for the plots against ρ, which are based on single-block exchangeable correlation matrix to make the plot against ρ meaningful).

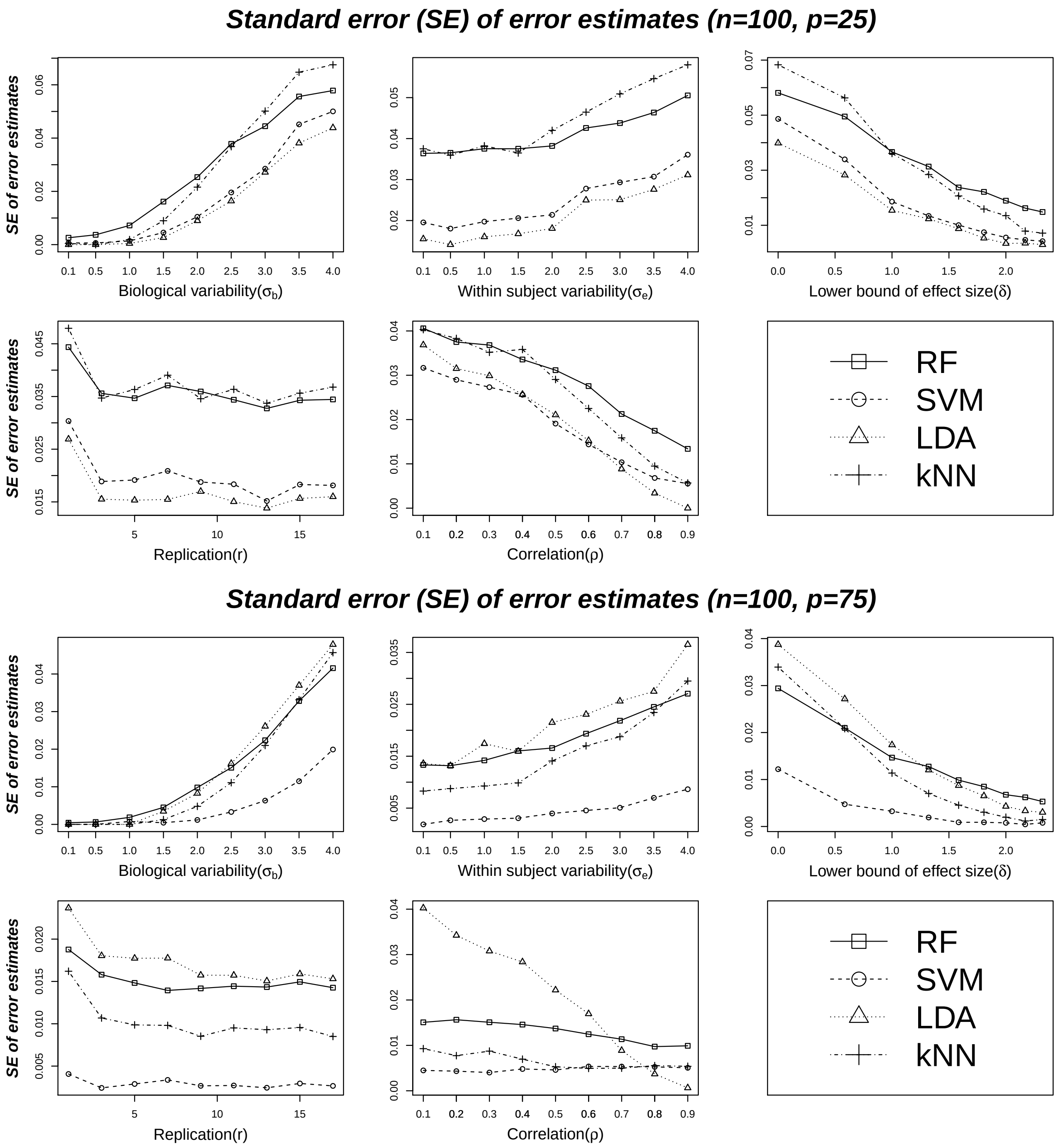

Figure S3: Standard errors of leave-one-out cross-validation error estimates at varying levels of different data characteristics. Top and bottom panels correspond to feature set sizes of 25 and 75 respectively and a common training sample size (n=100). Each plot compares SEs of leave-one-out error estimates for the four methods at varying levels of a particular parameter as shown on the x-axis for given values of the other parameters. The given values are selected from the set $(n = 100, \sigma_b = 2.5, \sigma_e = 1.5, \theta_{min} = 2, r = 3)$, the correlation structure being of the hub-Toeplitz form (except for the plots against ρ, which are based on single-block exchangeable correlation matrix to make the plot against ρ meaningful) For $p = 75$ SVM provides the most stable (lowest SE) estimates of leave-one-out error. LDA was found to give most precise estimates of error rates for $p = 25$ and higher correlations between features, but appears to be least stable for larger feature set size to sample size ratio $(p/n > 0.5)$. Strength of RF is visible in situations where data are more variable and have smaller effect sizes, in which cases it provides more stable error estimates than kNN and LDA.

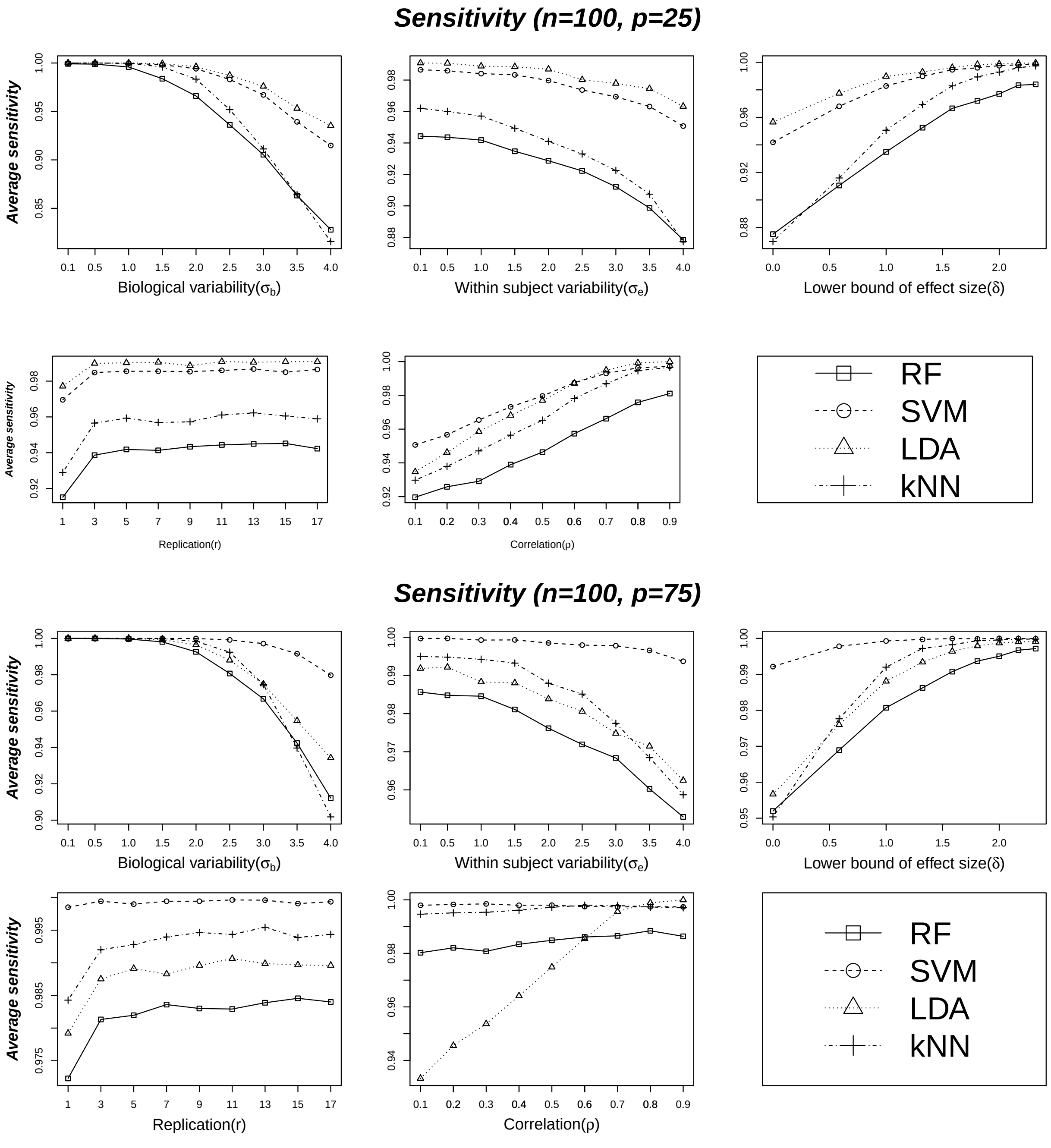

Figure S4: Average sensitivity at varying levels of different data characteristics: biological variability(σ_b), experimental noise (σ_e), lower bound of effect size (δ), replication (r), and correlation between variables (ρ). Top and bottom panels correspond to feature set sizes of 25 and 75 variables respectively and a common training set size (n=100). Each plot compares sensitivity for the four methods at varying levels of a particular parameter as shown on the x-axis for given values of the other parameters. The given values are selected from the set ($n = 100, \sigma_b = 2.5, \sigma_e = 1.5, \theta_{min} = 2, r = 3$), the correlation structure being of the hub-Toeplitz form (except for the plots against ρ, which are based on single-block exchangeable correlation matrix to make the plot against ρ meaningful).

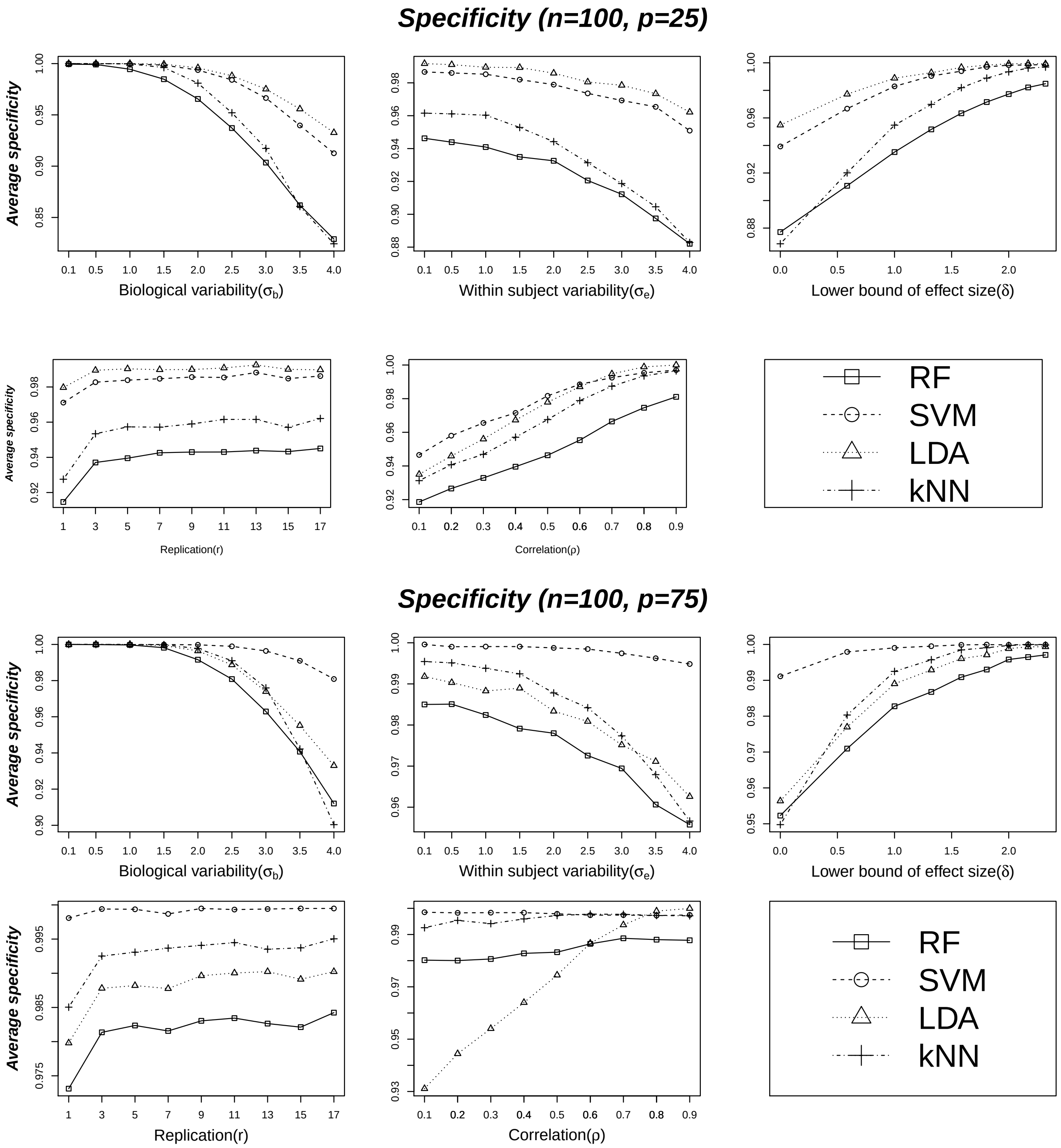

Figure S5: Average specificity at varying levels of different data characteristics: biological variability(σ_b), experimental noise (σ_e), lower bound of effect size (δ), replication (r), and correlation between variables (ρ). Top and bottom panels correspond to feature set sizes of 25 and 75 variables respectively and a common training set size (n=100). Each plot compares specificity for the four methods at varying levels of a particular parameter as shown on the x-axis for given values of the other parameters. The given values are selected from the set ($n = 100, \sigma_b = 2.5, \sigma_e = 1.5, \theta_{min} = 2, r = 3$), the correlation structure being of the hub-Toeplitz form (except for the plots against ρ, which are based on single-block exchangeable correlation matrix to make the plot against ρ meaningful).

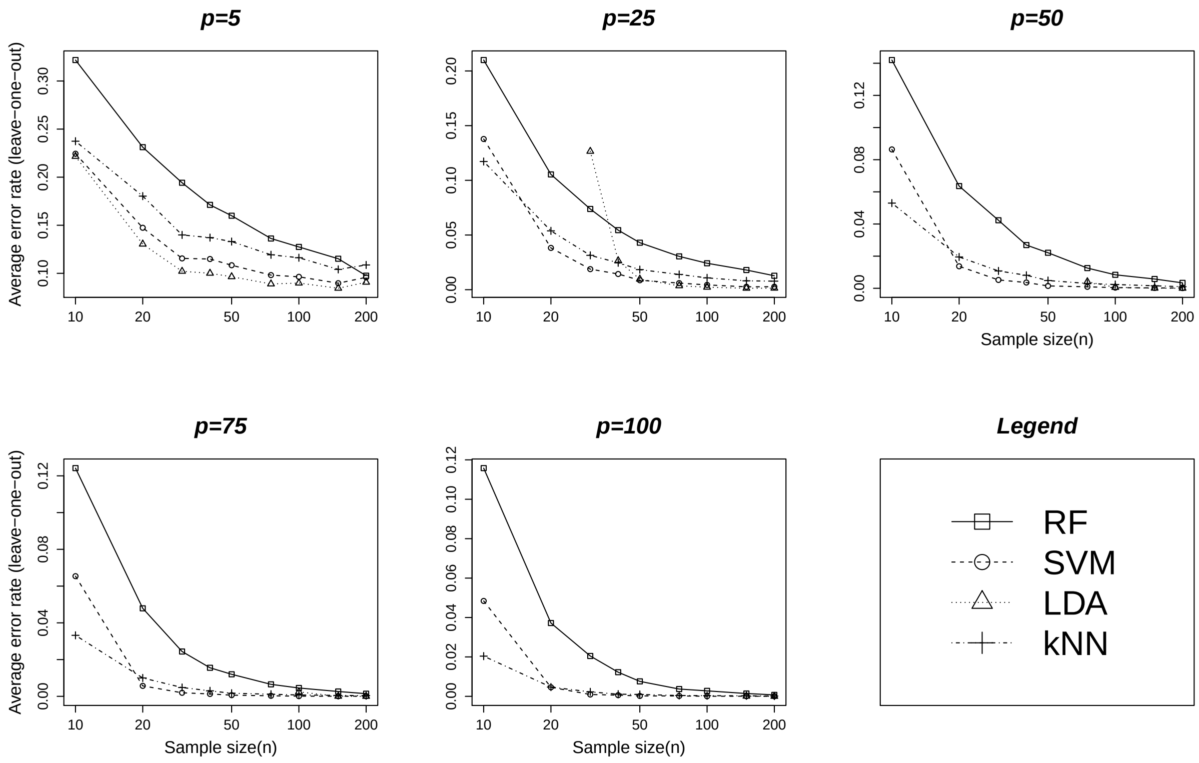

Figure S6: (Non-normal data) Average leave-one-out cross-validation error at varying levels of training sample and feature set sizes. Error rates are plotted against 9 different values of n as given in Table 1 (main paper). The five plots correspond to five different values of p (feature set size): 5, 25, 50, 75 and 100 respectively. The patterns and order of performances for Poisson data look very similar to that we observed for Gaussian data (Figure 2, main paper). Although SVM performs better than the other methods for larger feature set sizes (relative to sample size), the method can perform poorly if the sample size is too small ($n < 20$). LDA can theoretically handle feature set size as high as the sample size ($p < n$), but it's performance seem to deterirate as the fature set size (p) grows beyond approximately half the sample size (e.g., see the plot for $p = 25$).
